# Supplementary material for: Interaction between Acute Hepatic Injury and Early Coagulation Dysfunction on Mortality in Patients with Acute Myocardial Infarction
Source: J Clin Med. 2023 Feb 15;12(4):1534. doi: 10.3390/jcm12041534 (PMC9966038; doi:10.3390/jcm12041534)
Supplement: Supplementary file 1 [file jcm-12-01534-s001.zip › jcm-2138974-supplementary.pdf]

Table S1. Laboratory test data of AMI patients stratified by acute hepatic injury

Variables	Total (n=703)	Non-hepatic injury (n=596)	Hepatic injury (n=107)	P value
Coagulation function				
PLT (10^9/L)	209.0 (163.5-262.0)	211.5 (168.5-264)	192.0 (143.0-252.0)	0.354
APTT(s)	59.0 (32.7-100.4)	46.8 (31.9-94.4)	82.0 (38.4-139.8)	<0.001
INR	1.3 (1.1-1.5)	1.2 (1.1-1.4)	1.5 (1.3-2.4)	<0.001
PLT<150x10*9/L, n (%)	139 (19.772)	112 (18.792)	27 (25.234)	0.123
APTT>39s, n (%)	438 (65.766)	355 (63.167)	83 (79.808)	0.001
INR>1.4, n (%)	207 (31.175)	153 (27.224)	54 (52.941)	<0.001
Coagulation disorders, n (%)	502 (71.408)	411 (68.960)	91 (85.047)	<0.001
Liver function				
AST(U/L)	138.5 (60.0-259.5)	104.5 (50.0-178.5)	526.0 (250.5-815.0)	<0.001
ALP(U/L)	71.0 (55.0-94.0)	70.0 (55.0-88.0)	83.0 (59.0-105.5)	0.017
ALB(g/L)	3.4 (3.1-3.9)	3.6(3.1-3.9)	3.2(2.7-3.5)	<0.001
TBIL(mg/dL)	0.6 (0.4-0.9)	0.6 (0.4-0.9)	0.6 (0.5-1.0)	<0.001
Cardiac function				
CK(IU/L)	1431.0 (534.0-2930.5)	1080.0 (445.5-2037.5)	5206.5 (1630.0-7528.00)	<0.001
CK-MB(IU/L)	112.0 (29.5-239.0)	87.0 (25.0-188.0)	225.5 (105.0-386.0)	<0.001
LDH(IU/L)	466.0 (309.5-748.0)	404.0 (281.0-589.0)	1315.5 (666.0-1999.5)	<0.001

PLT, platelets; APTT, activated partial thromboplastin time; INR, international normalized ratio; AST, aspartate aminotransferase; ALP, alkaline phosphatase; ALB, albumin; TBIL, total bilirubin; CK, creatine kinase; CK-MB, creatine kinase-MB; LDH, lactate dehydrogenase.

Table S2. Outcomes of AMI patients stratified by acute hepatic injury and coagulation disorder.

Variables	Hepatic injury/ Coagulation disorder (n=91)	Hepatic injury/ Normal coagulation (n=16)	Non-hepatic injury/ Coagulation disorder (n=411)	Non-hepatic injury/ Normal coagulation (n=185)	P value
Length of hospital stay (Day)	6.915 (3.596-9.14.712)	5.352 (3.239-9.582)	5.958 (3.733-10.511)	3.924 (2.935-6.088)	<0.001
ICU stay (Day)	3.699 (1.860-7.715)	2.363 (1.153-5.509)	2.876 (1.536-5.576)	1.677 (1.167-2.841)	<0.001
Outcomes					
In-hospital mortality, n (%)	37 (40.659)	1 (6.250)	50 (12.165)	11 (5.946)	<0.001
ICU mortality, n (%)	36 (39.560)	1 (6.250)	40 (9.732)	9 (4.865)	<0.001
28-day mortality, n (%)	39 (42.857)	1 (6.250)	53 (12.895)	12 (6.486)	<0.001
90-day mortality, n (%)	43 (47.253)	3 (18.750)	75 (18.248)	12 (6.486)	<0.001

Table S3. Subgroup analysis for the associations between acute hepatic injury and outcomes

Predictors	Univariate regression analysis		Multivariate regression analysis	
	OR;95%CI	P value	OR;95%CI	P value
**PCI**				
In-hospital mortality	5.257 (2.844-9.718)	<0.001	3.812 (1.784-8.149)	<0.001
ICU mortality	6.375 (3.365-12.079)	<0.001	5.058 (2.281-11.220)	<0.001
D28 mortality	5.312 (2.894-9.752)	<0.001	3.856 (1.809-8.220)	<0.001
D90 mortality	5.143 (2.859-9.253)	<0.001	4.001 (1.931-8.290)	<0.001
PCI-Coagulation disorders				
In-hospital mortality	5.262 (2.705-10.238)	<0.001	4.243 (1.891-9.522)	<0.001
ICU mortality	6.733 (3.355-13.511)	<0.001	5.933 (2.487-14.153)	<0.001
D28 mortality	5.307 (2.746-10.258)	<0.001	4.228 (1.893-9.445)	<0.001
D90 mortality	4.692 (2.472-8.905)	<0.001	3.921 (1.801-8.537)	<0.001
PCI-Normal coagulation function				
In-hospital mortality	1.907 (0.206-17.631)	0.569	2.219 (0.036-137.371)	0.705
ICU mortality	1.907 (0.206-17.631)	0.569	2.219 (0.036-137.371)	0.705
D28 mortality	1.907 (0.206-17.631)	0.569	2.219 (0.036-137.371)	0.705
D90 mortality	4.292 (0.742-24.809)	0.104	8.415 (0.494-143.444)	0.141
**Anticoagulation/antiplatelet**				
In-hospital mortality	6.032 (3.355-10.846)	<0.001	4.385 (2.098-9.165)	<0.001
ICU mortality	6.804 (3.706-12.489)	<0.001	5.017 (2.322-10.840)	<0.001
D28 mortality	6.132 (3.459-10.871)	<0.001	4.692 (2.278-9.666)	<0.001
D90 mortality	5.190 (3.047-8.840)	<0.001	4.355 (2.235-8.484)	<0.001
Anticoagulation/antiplatelet -Coagulation disorders				

Supplementary table 3 continued

In-hospital mortality	6.710 (3.554-12.667)	<0.001	5.209 (2.373-11.434)	<0.001
ICU mortality	7.564 (3.916-14.610)	<0.001	6.350 (2.736-14.739)	<0.001
D28 mortality	6.730 (3.621-12.508)	<0.001	5.390 (2.516-11.547)	<0.001
D90 mortality	4.747 (2.665-8.454)	<0.001	4.211 (2.084-8.507)	<0.001
Anticoagulation/antiplatelet-Normal coagulation function				
In-hospital mortality	0.000	0.999	0.000	0.999
ICU mortality	0.000	0.999	0.000	0.999
D28 mortality	0.000	0.999	0.000	0.999
D90 mortality	3.543 (0.648-19.363)	0.144	10.782 (0.859-135.307)	0.065

Covariates of multivariate regression, including age, gender, log_2CKMB, ECI, anticoagulation, antiplatelet, PCI, CABG, thrombolysis, coagulation disorder

Table S4. Multivariate Regression of coagulation disorder related to outcomes stratified by acute hepatic injury

Predictors	Univariate regression analysis		Multivariate regression analysis	
	OR;95% CI	P value	OR;95% CI	P value
Total cohort				
Normal coagulation function	reference		reference	
In-hospital mortality	2.850 (1.581-5.139)	<0.001	2.673 (1.274-5.607)	0.009
ICU mortality	2.860 (1.521-5.380)	0.001	2.794 (1.241-6.288)	0.013
28-day mortality	2.832 (1.599-5.017)	<0.001	2.564 (1.261-5.213)	0.009
90-day mortality	3.419 (1.999-5.849)	<0.001	2.979 (1.545-5.743)	0.001
Hepatic injury				
Normal coagulation function	reference		reference	
In-hospital mortality	10.278 (1.301-81.206)	0.027	15.918 (1.384-183.140)	0.026
ICU mortality	9.818 (1.242-77.605)	0.030	12.124 (1.138-129.205)	0.039
28-day mortality	11.250 (1.425-88.832)	0.022	13.697 (1.432-131.023)	0.023
90-day mortality	3.882 (1.036-14.548)	0.044	4.033 (0.909-17.890)	0.067
Non-hepatic injury				
Normal coagulation function	reference		reference	
In-hospital mortality	2.191 (1.113-4.313)	0.023	1.899 (0.864-4.176)	0.111
ICU mortality	2.108 (1.001-4.441)	0.050	1.971 (0.823-4.721)	0.128
28-day mortality	2.314 (1.112-4.098)	0.023	1.779 (0.828-3.823)	0.140
90-day mortality	3.218 (1.703-6.081)	<0.001	2.716 (1.299-5.681)	0.008

Covariates of multivariate regression, including age, gender, log_2CKMB, ECI, anticoagulation, antiplatelet, PCI, CABG, thrombolysis, acute hepatic injury
